# CREB5 promotes nodal metastasis of cervical cancer by regulation of APLN-induced lymphangiogenesis

**DOI:** 10.1038/s41420-025-02782-5

**Published:** 2025-10-27

**Authors:** Meng Xia, Li Yuan, Linna Chen, Weijia Wen, Yan Jia, Xueyuan Zhao, Songlin Liu, Haolin Fan, Pan Liu, Hongye Jiang, Wei Wang, Yuandong Liao, Shuzhong Yao, Chunyu Zhang

**Affiliations:** 1 Department of Obstetrics and Gynecology, the First Affiliated Hospital, Sun Yat-sen University, Guangzhou, Guangdong P. R. China; 2 Guangdong Provincial Clinical Research Center for Obstetrical and Gynecological Diseases, Guangzhou, Guangdong P. R. China

**Keywords:** Cervical cancer, Tumour angiogenesis

## Abstract

Lymph node metastasis (LNM), a hallmark of aggressive cervical cancer (CCa), severely compromises prognosis and limits therapeutic efficacy. Despite its clinical significance, the molecular mechanisms driving LNM in CCa remain poorly characterized, necessitating urgent exploration. In this study, leveraging an in vitro lymphangiogenesis screening model, we identified CREB5 as a gene markedly upregulated in metastatic CCa, with its expression strongly correlating with LNM and poor patient survival. Mechanistic investigations revealed that CREB5 transcriptionally activates apelin (APLN) by directly binding to its canonical TGACG motif in the promoter region, thereby driving APLN-mediated lymphangiogenesis. Inhibition of CREB5 or treatment with ML221, a selective APLN receptor (APLNR) antagonist, potently suppressed lymphatic metastasis in preclinical CCa models. These findings establish CREB5 as a critical regulator of APLN-dependent lymphangiogenesis and LNM, highlighting its potential as a dual diagnostic biomarker and therapeutic target for mitigating lymphatic dissemination in CCa patients.

## Introduction

Cervical cancer (CCa) ranks as the fourth most prevalent malignancy and the fourth leading cause of cancer-related mortality among women globally, severely compromising patient survival and quality of life [1, 2]. A major contributor to poor therapeutic outcomes is the high prevalence of lymph node (LN) metastasis in CCa patients [3]. The 5-year survival rate plummets from 95% in localized cases to 33.3% in patients with lymph node metastasis (LNM) [3]. As pelvic LN metastasis emerges as a predominant driver of CCa mortality, elucidating the molecular mechanisms underlying LNM is critical to developing early diagnostic strategies and innovative therapies to combat metastatic progression.

The cAMP response element-binding 5 (CREB5), a member of the CREB protein family, binds cAMP response elements (CREs) with high affinity [4, 5]. CREB family members function as transcription regulators, governing key oncogenic processes, including proliferation, migration, invasion, stemness, and chemoresistance [6–10]. CREB5 has been implicated as an independent prognostic marker in ovarian cancer and a mediator of resistance to androgen deprivation therapy in prostate cancer [11–13]. However, its functional role and mechanistic contributions to cervical carcinogenesis, particularly in LN metastasis, remain poorly characterized.

Apelin (APLN), an endogenous ligand of the APJ receptor, is aberrantly overexpressed in diverse malignancies, including glioblastoma [14], esophageal cancer [15, 16], hepatocellular carcinoma [17], and prostate cancer [18]. Elevated APLN levels correlate with aggressive tumor behavior and adverse prognosis in endometrial and breast cancers [19, 20]. In CCa, APLN drives proliferation, migration, and glycolytic metabolism via the PI3K/AKT/mTOR pathway [21]. Building on this evidence, our study employs in vitro lymphangiogenesis assays and in vivo lymphatic metastasis models to demonstrate that CREB5 overexpression promotes LN metastasis in CCa. Mechanistically, CREB5 transcriptionally activates APLN expression, thereby inducing lymphangiogenesis in CCa both in vitro and in vivo. These findings establish CREB5 as a critical regulator of APLN-mediated lymphangiogenesis and metastatic spread, positioning it as a promising therapeutic target to mitigate LN metastasis in CCa patients.

## Results

### CREB5 correlates with lymph node metastasis in cervical cancer

**Fig. 1:**
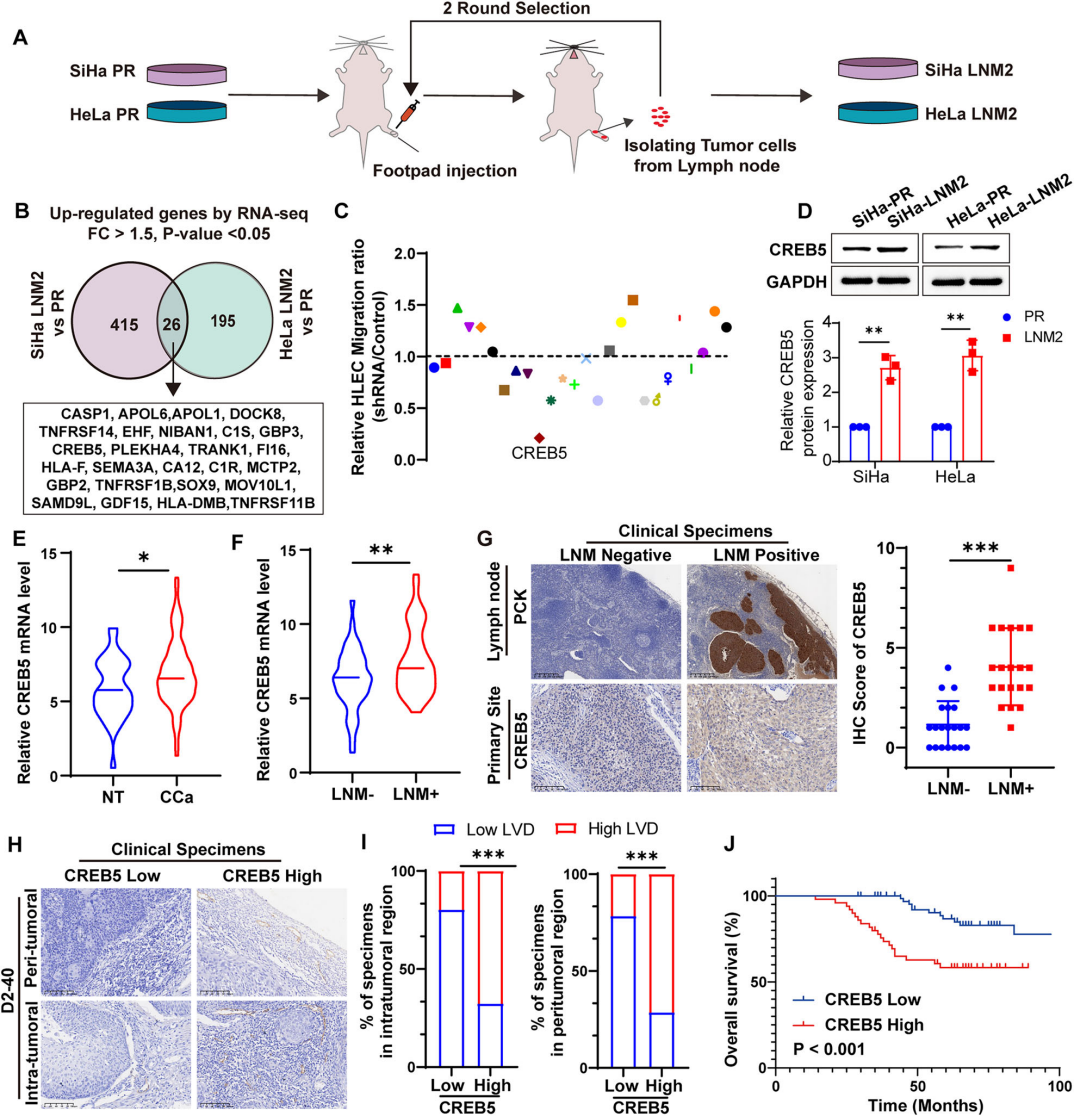
CREB5 is associated with lymph node metastasis in cervical cancer. **A** Schematic workflow of the screening strategy for identifying highly metastatic cervical cancer (CCa) cells. **B** Venn diagram identifying overlapping upregulated genes via RNA sequencing across the indicated groups. **C** Relative migration ratios of human lymphatic endothelial cells (HLECs) treated with conditioned medium from CCa cells transfected with shRNAs targeting 26 overlapping genes, compared to control cells. Data are normalized to the control group (set as 1.0). **D** Western blot analysis of CREB5 protein levels and quantification data in the indicated cell groups. GAPDH served as a loading control. **E** RT-qPCR quantification of CREB5 mRNA expression in normal cervical tissues (*n* = 33) versus CCa samples (*n* = 80). **F** RT-qPCR analysis of CREB5 expression in CCa tissues with lymph node metastasis (*n* = 40) versus LNM-negative tissues (*n* = 40). **G** Representative immunohistochemistry (IHC) images of CREB5 staining (left panel) and corresponding IHC scores (right panel) in LNM-positive versus LNM-negative CCa tissues. **H**, **I** Immunostaining of D2-40 (a lymphatic endothelial marker) in CREB5-low versus CREB5-high CCa tissues (representative images, **H**) and quantitative analysis of lymphatic vessel density (LVD; **I**). **J** Kaplan–Meier survival curves comparing overall survival in CCa patients stratified by CREB5 expression levels (high vs. low).

Our previous study established highly lymphatic metastatic CCa cells using a lymph node metastatic mouse model after two rounds of footpad injection [22]. In the present study, parental SiHa and HeLa cells were selected, as shown in the schematic in Fig. 1A. The parental SiHa and HeLa cells were inoculated into the footpad of immunodeficient mice, and metastatic cells were recovered from the popliteal LN. Since the parental SiHa and HeLa cells stably expressed a puromycin resistance element, these cells were screened by puromycin treatment, expanded in culture, and reinjected into mice for the next round of selection. After two rounds of selection, lymph node-metastatic subpopulations of SiHa and HeLa cells were established and designated as SiHa-LNM2 and HeLa-LNM2, respectively. To identify critical genes contributing to CCa LNM, RNA sequencing was performed using SiHa-LNM2 and SiHa-parental cells, as well as HeLa-LNM2 and HeLa-parental cells, revealing 26 upregulated genes in both groups (Fig. 1B). Given the importance of HLEC migration capability during lymphangiogenesis and LNM, we subjected the conditioned culture medium from shRNA-induced knockdown of the 26 genes to in vitro HLEC migration screening. We identified CREB5 as the most potent promoter of HLEC migration, enhancing the nodal metastasis of CCa cells (Fig. 1C). We then detected the expression levels of CREB5 protein in SiHa-LNM2 and SiHa-parental cells, as well as in HeLa-LNM2 and HeLa-parental cells. Results showed that CREB5 was the most upregulated one in SiHa-LNM2 and HeLa-LNM2 compared with their parental counterparts among the top 5 candidates selected based on the migration assay (Supplementary Fig. 1A, B). Consistently, CREB5 protein levels were significantly higher in SiHa-LNM2 and HeLa-LNM2 cells compared to parental cells (Fig. 1D). We further validated the expression levels of CREB5 mRNA and protein in CCa tissues. As shown in Fig. 1E, F, CREB5 mRNA levels were elevated in CCa tissues, particularly in those with LNM. Immunohistochemistry (IHC) revealed weak CREB5 protein expression in CCa tissues without LNM but strong expression in those with LNM (Fig. 1G). Subsequently, we enrolled a clinical cohort of 123 CCa cases and investigated the relationship between CREB5 expression and clinicopathological characteristics (Table 1). Higher CREB5 expression in primary sites was associated with lymphovascular space invasion (*P* < 0.001) and LNM (*P* < 0.001). Notably, higher CREB5 expression was accompanied by increased density of D2-40-marked microlymphatic vessels in both intratumoral and peritumoral regions (Fig. 1H, I), suggesting that CREB5 plays a significant role in LN metastasis and lymphangiogenesis in CCa. Furthermore, Kaplan–Meier survival analysis indicated that CCa patients with high CREB5 expression had significantly reduced overall survival (OS) (Fig. 1J). Collectively, these results suggest that CREB5 is positively associated with LNM in CCa.

**Table 1:** Correlation analyses between CREB5 and clinical parameters.

Variables	Total	CREB5 expression		*P* value
	123	Low	High	
Age				0.284
< 42	33	17	16	
≥ 42	90	56	34	
FIGO stage				0.196
I (IA2 + IB1 + IB2)	98	61	37	
II (IIA1 + IIA2)	25	12	13	
Tumor Size				0.508
≤ 4 cm	112	68	44	
> 4 cm	11	5	6	
Histopathologic types				0.179^a^
Squamous cell carcinoma	108	62	46	
Adenocarcinoma	9	8	1	
Adenosquamous carcinoma	6	3	3	
Differentiation				0.678^a^
High	1	1	0	
Medium	52	33	19	
Low	70	39	31	
Stromal invasion				0.115
< 1/2	42	29	13	
≥ 1/2	81	44	37	
LVSI				<0.001
Positive	51	4	47	
Negative	72	69	3	
LNM				<0.001
Positive	26	7	19	
Negative	97	66	31	

^a^Fisher’s exact test was applied.

### CREB5 promotes lymph node metastasis of CCa in vivo

**Fig. 2:**
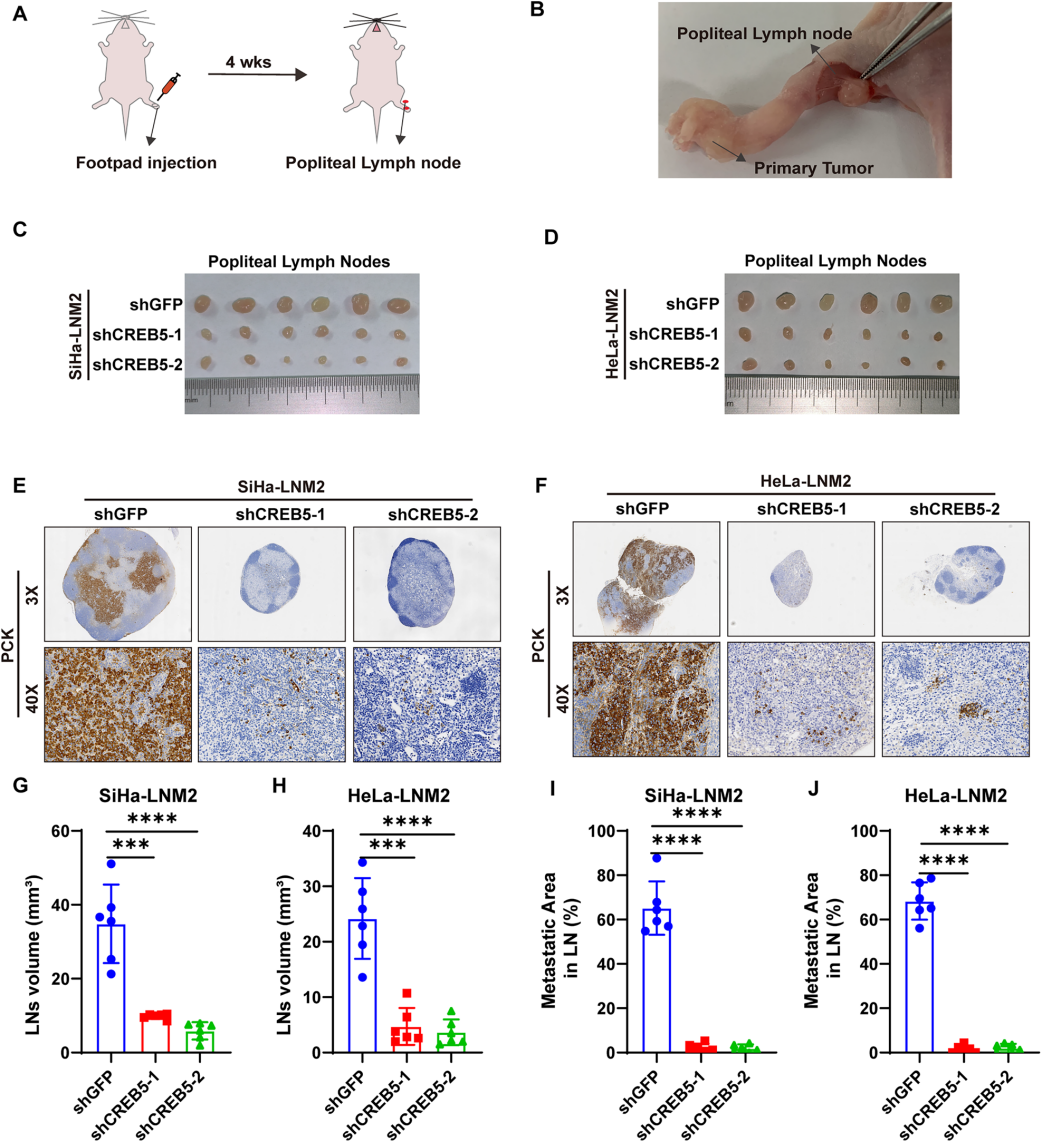
CREB5 drives lymph node metastasis of CCa in vivo. **A**, **B** Schematic illustration of the in vivo lymphatic metastasis model using nude mice. CCa cells stably expressing control (shGFP) or CREB5-targeting shRNA (shCREB5) were injected into the footpad of mice to assess popliteal LN metastasis. **C**, **D** Representative images of popliteal LNs harvested from shGFP and shCREB5 groups (*n* = 6 per group). **E**, **F** Immunostaining for pan-cytokeratin (a marker for epithelial cancer cells) in popliteal LNs, confirming metastatic CCa cells. **G**, **H** Quantitative analysis of popliteal LN volumes in the shGFP and shCREB5 groups. **I**, **J** Metastatic area within popliteal LNs.

To evaluate the impact of CREB5 on LNM in CCa in vivo, we employed a nude mouse LN metastasis model simulating directional lymphatic drainage and metastasis. We designed 2 shRNAs to silence CREB5, and the knockdown efficiency was demonstrated in Supplementary Fig. 3A. Tumor cells from different groups were implanted into the footpads of nude mice, and after 4 weeks, the popliteal LNs were collected and analyzed (Fig. 2A, B). Strikingly, silencing CREB5 notably suppressed LN metastasis of SiHa-LNM2 and HeLa-LNM2 cells (Fig. 2C, D). The volumes of the LNs were significantly smaller in the shCREB5-1 and shCREB5-2 groups compared to the control group (Fig. 2G, H). Pan-cytokeratin immunostaining confirmed that CREB5 ablation inhibited LNM of SiHa-LNM2 and HeLa-LNM2 cells (Fig. 2E, F and Supplementary Fig. 3B). Additionally, the metastatic area of the LNs was markedly reduced after CREB5 silencing (Fig. 2I, J).

### CREB5 facilitates lymphangiogenesis in CCa

**Fig. 3:**
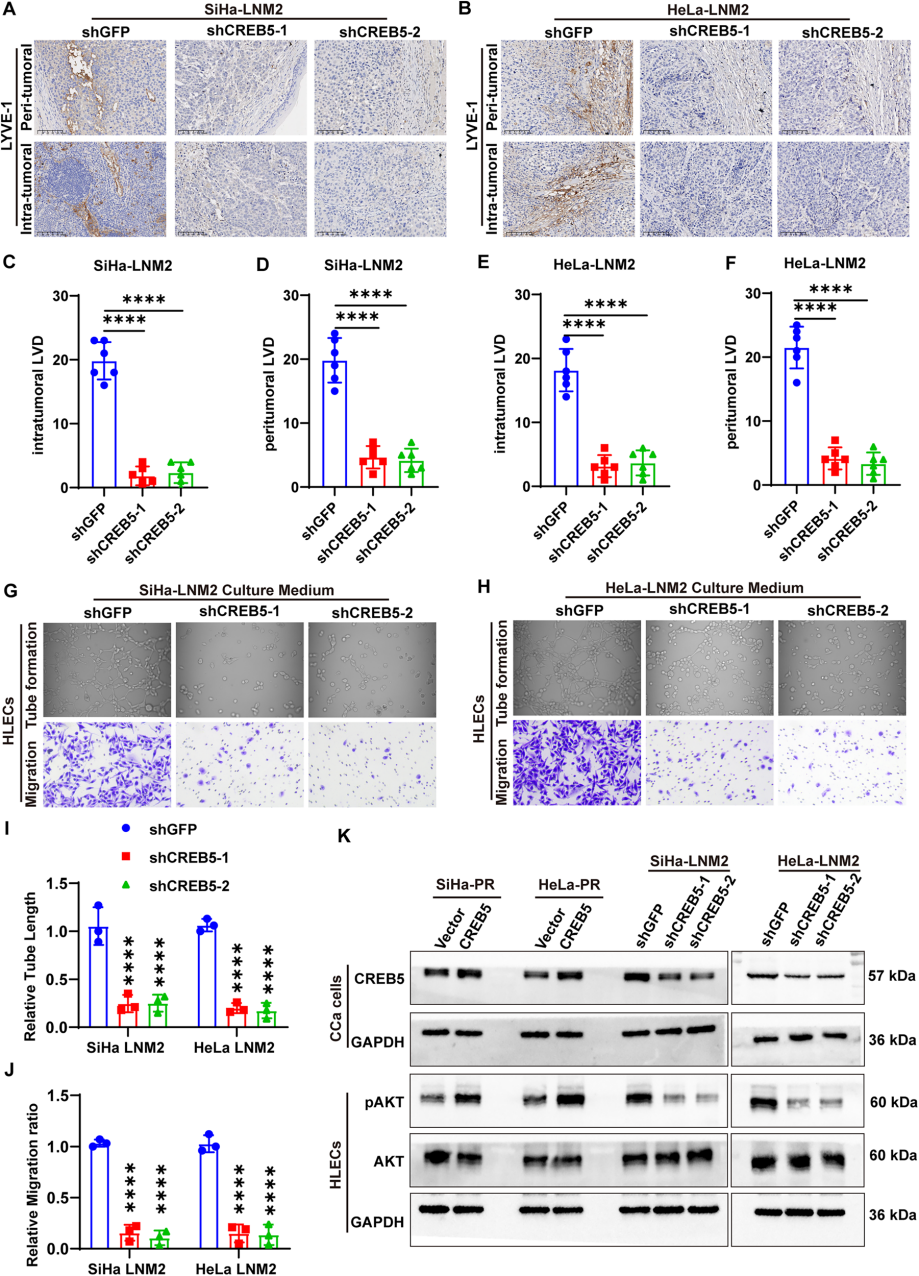
CREB5 promotes lymphangiogenesis in CCa. **A**, **B** Representative immunostaining images of D2-40 (lymphatic endothelial marker) in peritumoral and intratumoral regions of xenograft tumors from shGFP and shCREB5 groups. **C**–**F** Quantification of lymphatic vessel density (LVD) in peritumoral (**C**, **D**) and intratumoral (**E**, **F**) regions. **G**, **H** Representative images of tube formation (**G**) and transwell migration (**H**) assays for HLECs treated with conditioned medium from (CM) shGFP or shCREB5 CCa cells. **I**, **J** Quantitative analysis of tube length (**I**) and migrated HLECs (**J**). **K** Western blot analysis of total AKT and phosphorylated AKT (pAKT) in HLECs treated with CM from shCtrl or shCREB5 CCa cells.

As shown above, CREB5 was positively correlated with lymphovascular space invasion (LVSI) and lymphatic vessel density (LVD). We investigated whether CREB5 could influence lymphangiogenesis in CCa. We first confirmed the effective knockdown of CREB5 in primary tumors (Supplementary Fig. 3C). LYVE-1, a lymphatic vessel marker, was used to quantify intratumoral and peritumoral lymphatic vessels in primary tumors via IHC (Fig. 3A, B). Interestingly, lymphatic vascular density in mice inoculated with shRNA-CREB5 cells was reduced compared to mice bearing shGFP cells in both intratumoral and peritumoral regions (Fig. 3C–F), suggesting that CREB5 promotes lymphangiogenesis in vivo. To further elucidate how CREB5 promotes lymphangiogenesis in vitro, we collected conditioned media (CM) from CREB5-knockdown and control CCa cells to treat human lymphatic endothelial cells (HLECs). Conditioned media from CREB5-knockdown SiHa-LNM2 and HeLa-LNM2 cells significantly impaired tube formation and migration of HLECs compared to controls (Fig. 3G–J). These findings collectively indicate that CREB5 facilitates lymphangiogenesis in CCa both in vitro and in vivo. Additionally, conditioned media from CREB5-overexpressing cells significantly increased, while those from CREB5-knockdown cells markedly attenuated, the expression of p-AKT in HLECs, a crucial pathway in lymphangiogenesis (Fig. 3K). In addition to validating the effects on lymphangiogenesis, we also compared the impact of CREB5 on CCa cells’ migration and Epithelial-Mesenchymal Transition (EMT) ability. We found that knocking down CREB5 inhibited migration and concurrently reduced vimentin expression, suggesting that CREB5 not only promoted lymphangiogenesis but also enhanced the malignant potential of CCa cells themselves (Supplementary Fig. 2A–C).

### CREB5 promotes transcription of the target gene APLN

**Fig. 4:**
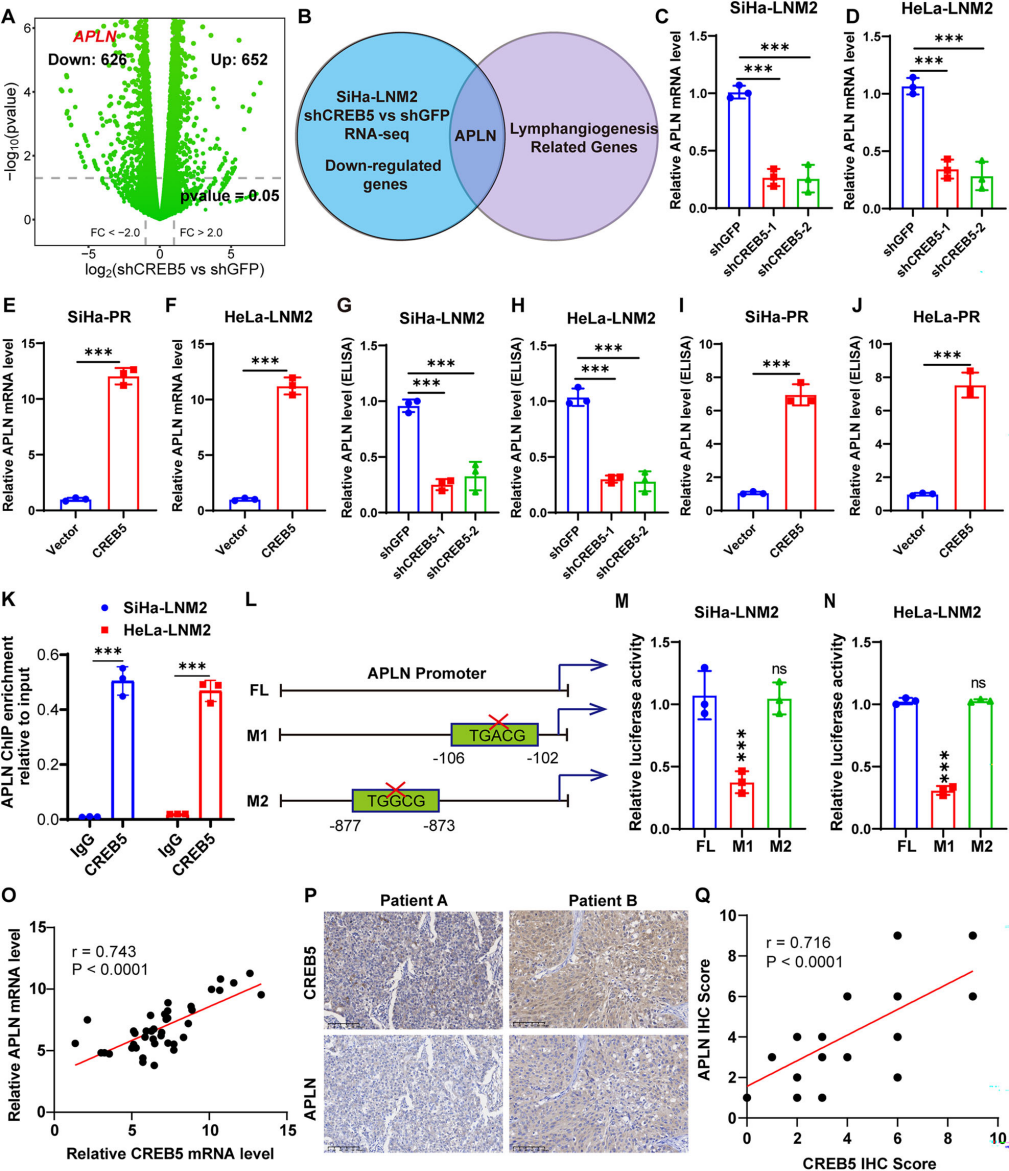
CREB5 transcriptionally activates APLN expression. **A** Volcano plot illustrating differentially expressed genes following CREB5-knockdown (shCREB5) compared to control (shGFP) in CCa cells. **B** Venn diagram identifying the overlap between CREB5-knockdown-induced downregulated genes in CCa cells and lymphangiogenesis-associated genes. **C**–**F** RT-PCR analysis demonstrating CREB5-dependent modulation of APLN mRNA levels in indicated cell lines. **G**–**J** ELISA quantification confirming CREB5-mediated regulation of secreted APLN protein in corresponding cell models. **K** ChIP assay validating direct binding of CREB5 to the APLN promoter in SiHa-LNM2 and HeLa-LNM2 cells (IgG as negative control). **L** Schematic of APLN promoter luciferase reporter constructs with wild-type (WT), canonical motif (TGACG), and non-canonical motif (TGGCG) deletions. **M**, **N** Luciferase reporter assays showing CREB5-dependent transcriptional activation of APLN promoter activity in SiHa-LNM2 and HeLa-LNM2 cells. **O** Positive correlation between CREB5 and APLN mRNA expression in CCa patient specimens. **P**, **Q** Representative immunohistochemical images and correlation analysis of CREB5 and APLN protein expression in CCa tissues.

Given that CREB is a key transcription factor family involved in regulating gene expression related to autophagy, ROS production, mitochondrial function, and antioxidant response [23], we sought to identify the transcriptional target of CREB5 in CCa. RNA sequencing was performed using shCREB5 and shGFP cells (Fig. 4A). We overlapped downregulated genes from RNA-seq upon CREB5-knockdown with lymphangiogenesis-related genes and identified APLN as the sole target mediating CREB5-induced lymphangiogenesis in CCa (Fig. 4B). In addition, we found APLN was significantly upregulated in SiHa-LNM2 and HeLa-LNM2 compared with SiHa-PR and HeLa-PR, respectively (Supplementary Fig. 4A). Subsequent qRT-PCR analysis revealed that APLN expression was significantly reduced in CREB5-knockdown SiHa-LNM2 and HeLa-LNM2 cells, while markedly increased in CREB5-overexpressing SiHa-PR and HeLa-PR cells (Fig. 4C–F). Similar results were observed in ELISA assays (Fig. 4G–J). Given that CREB5 is a transcription factor, we hypothesized that CREB5 might promote APLN transcription. Chromatin immunoprecipitation (ChIP) assays confirmed that CREB5 interacts with the APLN promoter (Fig. 4K). Furthermore, since CREB5 binding sites include the canonical motif (TGACG) and the non-canonical motif (TGGCG) [24], and the APLN promoter contains one canonical and one non-canonical motif, we constructed two APLN deletion mutation vectors (M1 and M2) based on these binding sites (Fig. 4L). Dual-luciferase reporter assays revealed that only the M1 vector exhibited significantly lower luciferase activity compared to the wild-type (FL) in both SiHa-LNM2 and HeLa-LNM2 cells (Fig. 4M, N), indicating that CREB5 binds to the TGACG motif in the APLN promoter. Additionally, we found a positive correlation between CREB5 and APLN mRNA and protein expression levels in CCa tissues (Fig. 4O–Q). In summary, we demonstrated that APLN is a transcriptional target of CREB5 in CCa lymphangiogenesis and lymphatic metastasis.

### CREB5 promotes CCa lymph node metastasis and lymphangiogenesis through APLN

**Fig. 5:**
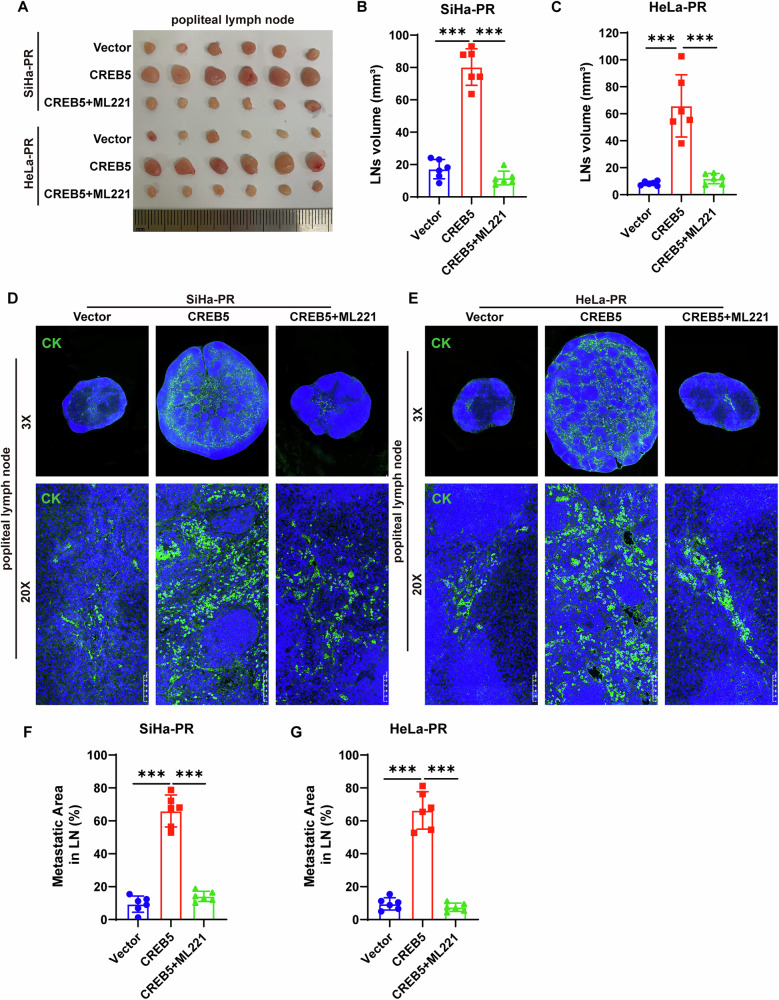
CREB5 promotes cervical carcinoma nodal metastasis through an APLN-dependent mechanism. **A** Representative macroscopic images of popliteal lymph nodes from different experimental groups (*n* = 6 per group). **B**, **C** Quantitative analysis of lymph node volume across different groups. **D**, **E** Immunofluorescence staining for pan-cytokeratin in popliteal LNs from different treatment groups. **F**, **G** Comparative analysis of metastatic burden in popliteal LNs from SiHa-PR and HeLa-PR cell treatment groups.

**Fig. 6:**
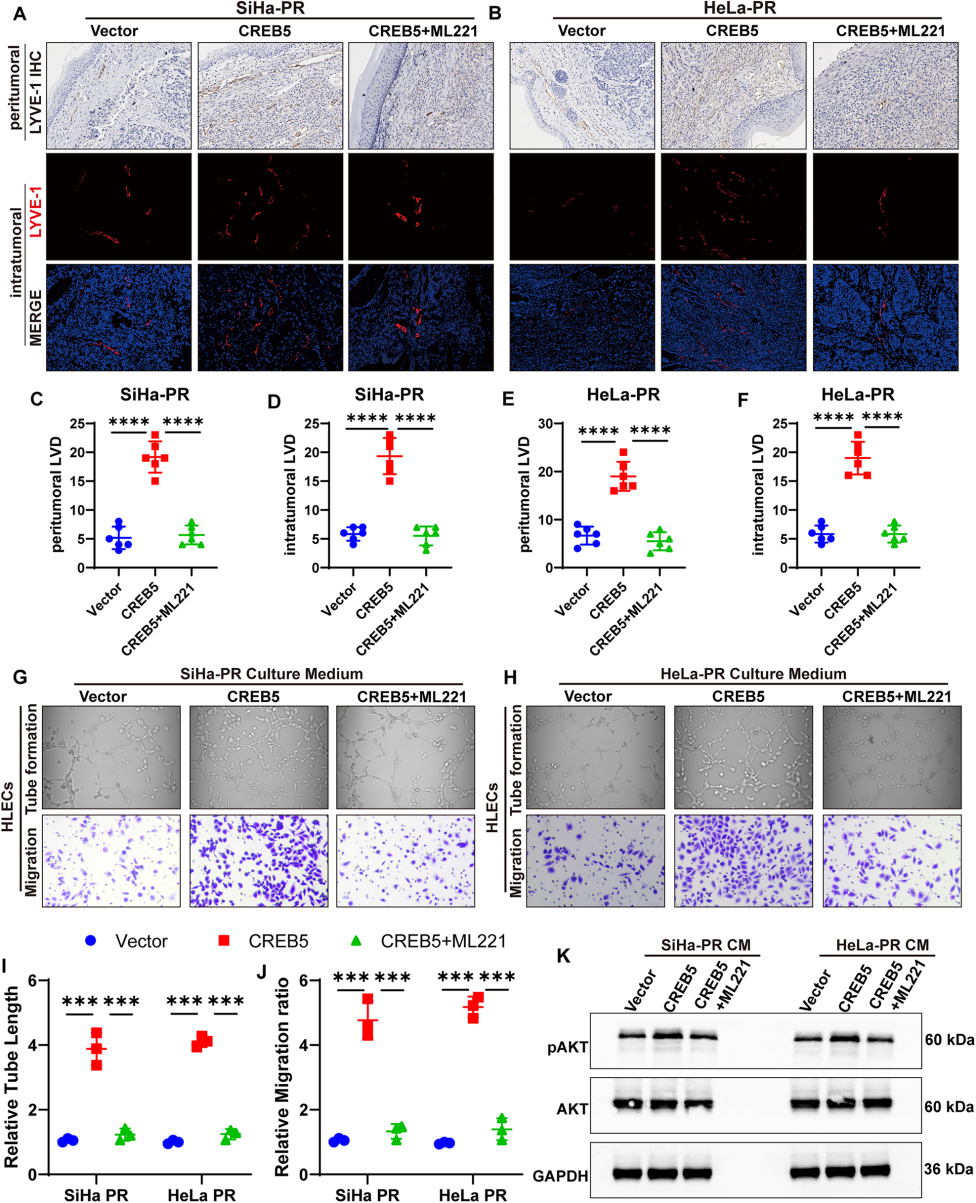
CREB5 facilitates cervical carcinoma-associated lymphangiogenesis through APLN-dependent mechanisms. **A**, **B** Representative IHC and IF images demonstrating LYVE-1-positive lymphatic vessels in peritumoral and intratumoral regions across experimental groups. **C**–**F** Quantitative assessment of lymphatic vascular density (LVD) in peritumoral and intratumoral microenvironments among treatment groups. **G**, **H** In vitro lymphangiogenic potential assessed by tube formation assays (**G**) and Transwell migration assays (**H**) using human lymphatic endothelial cells (HLECs) exposed to conditioned media from indicated CCa cells. **I**, **J** Quantitative analysis of tube length (**I**) and migrated HLECs in indicated groups (**J**). **K** Western blot analysis of total AKT and pAKT in HLECs treated with CM from different CCa cells.

To elucidate the role of APLN in CREB5-mediated LNM in CCa, we employed ML221 [25], a potent APLNR functional antagonist, in rescue experiments. Inhibition of APLN in SiHa-PR and HeLa-PR cells significantly reversed the promoting effect of CREB5 overexpression on LNM in vivo (Fig. 5A). Additionally, CREB5 overexpression significantly increased the volume of nude mice LNs, while ML221 reversed this effect (Fig. 5B, C). Immunofluorescence staining for cytokeratin confirmed that forced expression of CREB5 significantly enhanced the lymphatic metastatic ability of parental SiHa and HeLa cells, which was reversed by ML221 treatment (Fig. 5D, G). Similarly, ablation of APLN reduced intratumoral and peritumoral lymphatic vascular density in CREB5-overexpressing mice (Fig. 6A–F). Concordantly, tube formation and migration of HLECs incubated with conditioned media from CREB5-overexpressing SiHa-PR and HeLa-PR cells were markedly reversed by ML221 treatment (Fig. 6G, I). Furthermore, attenuation of APLN compromised the expression of p-AKT in HLECs after incubation with conditioned media from CREB5-overexpressing CCa cells (Fig. 6K). These findings indicate that APLN is essential for CREB5 to promote LNM in CCa.

## Discussion

**Fig. 7:**
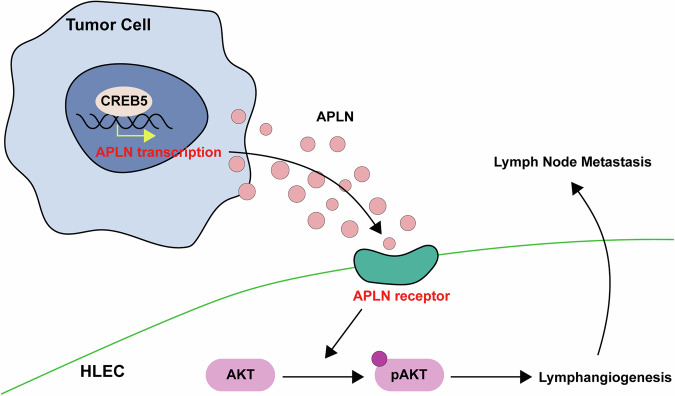
A schematic diagram of the mechanism.

In this study, we identified CREB5 as a critical regulator of LNM in CCa, driven by its transcriptional upregulation of apelin (APLN), which subsequently promotes lymphangiogenesis (Fig. 7). Our findings elucidate novel molecular mechanisms underlying lymphatic metastasis in CCa and propose CREB5 and APLN as potential therapeutic targets for mitigating metastatic progression.

CREB5, a member of the cAMP response element-binding (CREB) protein family, has been implicated in tumor proliferation, migration, and invasion [6, 9, 26]. However, its role in LNM remains poorly characterized. Through comparative analysis of differentially expressed genes between LN-metastatic CCa cells and their parental counterparts, combined with in vitro HLECs migration assays, we demonstrate for the first time that CREB5 is a pivotal regulator of LNM in CCa. Clinically, CREB5 expression was significantly elevated in CCa patients with LNM compared to those without LNM, and high CREB5 levels correlated with reduced OS. Furthermore, CREB5 expression positively associated with LVSI and lymphatic vessel density, both critical determinants of metastatic dissemination. Functionally, CREB5 overexpression in CCa cells increased HLECs tube formation and migration via conditioned media, and activated the pAKT pathway in HLECs, a key mediator of lymphangiogenesis [27, 28]. These data collectively suggest that CREB5 facilitates LNM by enhancing lymphangiogenesis, thereby promoting metastatic spread.

APLN, a peptide hormone linked to angiogenesis and lymphangiogenesis [29–31], has been reported to drive cancer progression in multiple malignancies. For instance, APLN enhances breast cancer cells proliferation and invasion [32], sustains vascularization in glioblastoma (GBM) [33], and correlates with vascular invasion in bladder cancer [34]. Notably, APLN overexpression amplifies intratumoral lymphangiogenesis and LNM [31]. Here, we identified APLN as a direct transcriptional target of CREB5 in CCa. ChIP assays confirmed CREB5 binding to the canonical TGACG motif within the APLN promoter, driving its expression. APLN, in turn, mediated CREB5-induced lymphangiogenesis and LNM. Critically, the APLN receptor antagonist ML221 abolished the pro-metastatic effects of CREB5 overexpression in animal models, underscoring the therapeutic potential of targeting APLN signaling in metastatic CCa.

In conclusion, our study establishes a CREB5-APLN regulatory axis as a central driver of LNM in CCa, mediated through promoting lymphangiogenesis. These findings advance the mechanistic understanding of lymphatic metastasis and nominate CREB5 and APLN as actionable therapeutic targets. Future research should prioritize the development of small-molecule inhibitors or antibody-based strategies to disrupt this axis, offering new avenues to improve clinical outcomes in metastatic CCa patients.

## Materials and Methods

### Cell culture

In this research, human CCa cell lines (SiHa and HeLa) were utilized, all procured from the American Type Culture Collection (ATCC). HeLa LNM2 and SiHa-LNM2 cells were established as previously reported [22]. These cells were maintained in DMEM (Gibco, USA) supplemented with 10% fetal bovine serum (FBS) (Corning, USA), 1% non-essential amino acids (Corning, USA), and 1% penicillin-streptomycin (Gibco, USA). HLECs were acquired from Otwo Biotech and grown in Endothelial Cell Medium (ScienCell). All cell lines were incubated at 37 °C in a humidified atmosphere containing 5% CO_2_. The authenticity of these cell lines was verified through short tandem repeat genotyping, and mycoplasma contamination was assessed using the e-Myco Mycoplasma PCR Detection Kit (iNtRON). Experiments were conducted using cells in the logarithmic growth phase.

### Patients and specimens

Fresh CCa tissue samples were collected from patients who underwent radical hysterectomy and lymphadenectomy between 2018 and 2023 at the First Affiliated Hospital of Sun Yat-sen University (Guangzhou, China). Before samples were collected, each patient provided signed informed consent. Immediately after surgical removal, the fresh specimens were snap-frozen in liquid nitrogen and stored at −80 °C until further use. None of the included patients had received radiotherapy or chemotherapy prior to surgery. Normal cervical tissues were collected from patients who underwent hysterectomy for benign gynecological conditions. This study was approved by the Ethical Review Committee of the First Affiliated Hospital of Sun Yat-sen University (approval number: 2023-008). All procedures involving human participants were conducted in compliance with the Declaration of Helsinki.

### Plasmid construction and transfection, lentivirus production, and transduction

The shCREB5 plasmid and CREB5 overexpression plasmid were synthesized by Tsingke Biotechnology Co., Ltd. These plasmids were co-transfected with psPAX2 and pMD2.G into 293T cells using X-tremeGENE HP DNA transfection reagent (Roche, Mannheim, Germany) to produce lentiviral particles. Following transfection, the lentivirus-containing supernatant was collected. Lentiviral transduction was performed according to the manufacturer’s protocol, with the addition of polybrene at a final concentration of 10 μg/ml to enhance transduction efficiency. To establish stable cell lines, transduced cells were selected using puromycin (2 μg/ml, Sigma-Aldrich, USA) for 7 days post-transduction.

### Enzyme-linked immunosorbent assay

Enzyme-linked immunosorbent assay (ELISA) kits (Thermo Fisher Scientific Shier Technology, United States) were utilized to measure the levels of APLN in indicated cells in accordance with the manufacturer’s instructions.

### Chromatin immunoprecipitation (ChIP) assay

Cells were fixed with 37% formaldehyde at room temperature for 15 min. Subsequently, the DNA was fragmented into segments ranging from 500 to 1000 base pairs using sonication. The chromatin was then immunoprecipitated using an anti-CREB5 antibody, with IgG serving as a negative control, following the protocol provided with the DNA Purification and ChIP Assay Kit. ChIP-PCR primers specific to the APLN gene were designed and synthesized by GENEWIZ, China. These primers were used to quantify APLN expression via qPCR after chromatin purification.

### Dual-luciferase reporter assay

Cells transfected with a CREB5 overexpression plasmid were co-transfected with a luciferase reporter plasmid (200 ng/well) and the Renilla luciferase control vector pRL-CMV (10 ng/well; Hanyin Biotech, China). At 24 h post-transfection, luciferase activity was analyzed using the Dual-Luciferase Reporter Assay Kit (Beyotime, Shanghai, China) in accordance with the manufacturer’s protocol.

### HLECs transwell assay and tube formation assay

For transwell migration assays, 3 × 10^4^ cells/500 μL were seeded into the upper chamber with serum-free culture medium, while the lower chamber was 500 μL complete medium with 15% FBS of indicated CCa cells. After 48 h, the cells on the lower surface of the chamber were fixed with paraformaldehyde and then stained with crystal violet. The numbers of migrated cells were counted under microscope in 5 random fields. For HLECs tube formation assay, HLECs at the density of 1 × 10^4^ cells/well were seeded onto 96-well plates with cell culture medium of indicated CCa cells, which had been precoated with Matrigel and incubated under 37 °C for 6 h. Tube formation was quantified by measuring the relative tube length in 3 random fields under a light microscope and Image J was used to process these data.

### Immunohistochemistry (IHC) and immunofluorescence (IF) staining

Briefly, 5 μm thick paraffin-embedded tissue sections were deparaffinized and rehydrated. After rinsing with PBS, the sections were subjected to antigen retrieval by heating in 0.01 mol L^−1^ sodium citrate buffer (pH 6.0), followed by washing with PBS. The sections were then blocked with 5% goat serum to prevent non-specific binding, followed by incubation with a primary antibody overnight at 4 °C. Subsequently, the sections were incubated with an HRP-conjugated secondary antibody for 1 h at room temperature. The staining process was completed using DAB for detection, hematoxylin for counterstaining, and dehydration and mounting in the appropriate sequence. Two experienced pathologists independently evaluated and scored the IHC staining results in a blinded fashion. The intensity of IHC staining (I) was graded as 0 (negative), 1 (weak), 2 (moderate), or 3 (strong). The percentage of positively stained cells (P) was categorized as 0 (≤ 25%), 1 (26–50%), 2 (51–75%), or 3 (≥ 76%). The IHC score (Q) was calculated by multiplying the staining intensity score (I) by the percentage score (P), i.e., Q = P × I. For IF, tissue sections were deparaffinized in xylene and rehydrated through a graded ethanol series. After rinsing with PBS, the sections were subjected to antigen retrieval by heating in 0.01 mol L^−1^ sodium citrate buffer (pH 6.0), followed by washing with PBS. Non-specific binding was blocked by incubating the tissues with 5% donkey serum. The sections were then incubated overnight at 4 °C with the appropriate primary antibodies. The next day, after several washes, the tissues were incubated for 30 min at room temperature with the corresponding secondary antibodies. Immunofluorescent images were captured using an LSM880 confocal microscope (Carl Zeiss). The antibodies used in this study are listed in Supplementary Table S1. All the uncropped blots used in the study were uploaded in Supplementary Material.

### RNA extraction, reverse transcription (RT), and qPCR

Total RNA was isolated from cultured cells or tissues using the SteadyPure Quick RNA Extraction Kit (Accurate Biology) following the manufacturer’s protocol. RNA concentration was measured using a Nanodrop2000 (Thermo Fisher Scientific), and equal amounts of RNA were reverse transcribed into cDNA using the Evo M-MLV RT-PCR Kit (Accurate Biology) according to the manufacturer’s instructions. The resulting cDNA was then subjected to real-time RT-PCR using 2× SYBR® Green Pro Taq HS Premix (Accurate Biology). Gene expression data were normalized to the geometric mean of the housekeeping gene GAPDH and analyzed using the 2^−ΔΔCT^ method. The primer pairs specific for various genes used in our experiments included: GAPDH Forward: 5′-AGAAGGCTGGGGCTCATTTG-3′, GAPDH Reverse: 5′-AGGGGCCATCCACAGTCTTC-3′; CREB5 Forward: 5′-CCCTGCCCAACCCTACAATG-3′, CREB5 Reverse: 5′-GGACCTTGCATCCCCATGAT-3′; APLN Forward: 5′-GTCTCCTCCATAGATTGGTCTGC-3′, APLN Reverse: 5′-GGAATCATCCAAACTACAGCCAG-3′.

### Animal experiments

Female BALB/c nude mice (4–6 weeks old, 18–22 × *g*) were procured from the Experimental Animal Center of Sun Yat-sen University and maintained under specific pathogen-free conditions. Mice were randomly assigned to experimental groups using a simple randomization method, with no blinding implemented during the study. For the footpad implantation model, CCa cells (2 × 10^6^/50 μL) were subcutaneously injected into the footpad region. Mice were euthanized and popliteal LNs were surgically excised for subsequent analyses at the experimental endpoint. ML221 (5 mg/kg) or placebo was administered via intraperitoneal injection every other day, starting when palpable tumors were detected at the inoculation site. At the experimental endpoint, mice were anesthetized and euthanized, followed by the removal of primary footpad tumors and associated popliteal LNs. LN volumes were calculated using the formula: Volume(mm^3^) = (length[mm]) × (width[mm])^2^ × 0.52. Excised primary tumors and LNs were fixed, paraffin-embedded, and processed for IHC or IF staining. All procedures strictly adhered to institutional guidelines and were approved by the Sun Yat-sen University Institutional Animal Care and Use Committee (approved number: SYSU-IACUC-2025-000011).

### Statistical analysis

GraphPad Prism Version 9.0 was utilized for generating graphs and performing statistical analyses in this study. Differences between groups were assessed using the two-tailed Student’s *t*-test. OS was analyzed using the Kaplan–Meier method, and the curves were compared using the log-rank test. The associations between CREB5 expression and clinicopathological factors were evaluated using the Pearson χ2 test or Fisher’s exact test, as appropriate. Correlations were analyzed using Pearson’s correlation coefficient. Data are presented as mean ± SD, with error bars representing the standard deviation. Statistical significance was defined as *p < 0.05, **p < 0.01, ***p < 0.001, and ****p < 0.0001.

## Supplementary information

supplementary figures and figure legends.

supplementary Table S1

uncropped blots

## Data Availability

All data are available from the corresponding author upon reasonable request.

## References

[1] Sung H, Ferlay J, Siegel RL, Laversanne M, Soerjomataram I, Jemal A, et al. Global cancer statistics 2020: GLOBOCAN estimates of incidence and mortality worldwide for 36 cancers in 185 countries. CA Cancer J Clin. 2021;71:209–49. 33538338 10.3322/caac.21660

[2] Bray F, Ferlay J, Soerjomataram I, Siegel RL, Torre LA, Jemal A. Global cancer statistics 2018: GLOBOCAN estimates of incidence and mortality worldwide for 36 cancers in 185 countries. CA Cancer J Clin. 2018;68:394–424. 30207593 10.3322/caac.21492

[3] Fleming ND, Frumovitz M, Schmeler KM, dos Reis R, Munsell MF, Eifel PJ, et al. Significance of lymph node ratio in defining risk category in node-positive early stage cervical cancer. Gynecol Oncol. 2015;136:48–53. 25451695 10.1016/j.ygyno.2014.11.010 PMC4430191

[4] Nomura N, Zu YL, Maekawa T, Tabata S, Akiyama T, Ishii S. Isolation and characterization of a novel member of the gene family encoding the cAMP response element-binding protein CRE-BP1. J Biol Chem. 1993;268:4259–66. 8440710

[5] Conkright MD, Montminy M. CREB: the unindicted cancer co-conspirator. Trends Cell Biol. 2005;15:457–9. 16084096 10.1016/j.tcb.2005.07.007

[6] Dai W, Hong L, Xiao W, Zhang L, Sha W, Yu Z, et al. The ATF2/miR-3913-5p/CREB5 axis is involved in the cell proliferation and metastasis of colorectal cancer. Commun Biol. 2023;6:1026. 37816820 10.1038/s42003-023-05405-w PMC10564889

[7] Tong T, Qin X, Jiang Y, Guo H, Wang X, Li Y, et al. A novel CREB5/TOP1MT axis confers cisplatin resistance through inhibiting mitochondrial apoptosis in head and neck squamous cell carcinoma. BMC Med. 2022;20:231. 35773668 10.1186/s12916-022-02409-x PMC9248137

[8] Kim HJ, Jeon HM, Batara DC, Lee S, Lee SJ, Yin J, et al. CREB5 promotes the proliferation and self-renewal ability of glioma stem cells. Cell Death Discov. 2024;10:103. 38418476 10.1038/s41420-024-01873-z PMC10901809

[9] Wang S, Qiu J, Liu L, Su C, Qi L, Huang C, et al. CREB5 promotes invasiveness and metastasis in colorectal cancer by directly activating MET. J Exp Clin Cancer Res. 2020;39:168. 32843066 10.1186/s13046-020-01673-0 PMC7446182

[10] Wu Z, Wang X, Wu H, Du S, Wang Z, Xie S, et al. Identification of CREB5 as a prognostic and immunotherapeutic biomarker in glioma through multi-omics pan-cancer analysis. Comput Biol Med. 2024;173:108307. 38547657 10.1016/j.compbiomed.2024.108307

[11] He S, Deng Y, Liao Y, Li X, Liu J, Yao S. CREB5 promotes tumor cell invasion and correlates with poor prognosis in epithelial ovarian cancer. Oncol Lett. 2017;14:8156–61. 29250192 10.3892/ol.2017.7234 PMC5727587

[12] Hwang JH, Arafeh R, Seo JH, Baca SC, Ludwig M, Arnoff TE, et al. CREB5 reprograms FOXA1 nuclear interactions to promote resistance to androgen receptor-targeting therapies. Elife. 2022;11:e73223. 35550030 10.7554/eLife.73223 PMC9135408

[13] Hwang JH, Seo JH, Beshiri ML, Wankowicz S, Liu D, Cheung A, et al. CREB5 promotes resistance to androgen-receptor antagonists and androgen deprivation in prostate cancer. Cell Rep. 2019;29:2355–70. 31747605 10.1016/j.celrep.2019.10.068 PMC6886683

[14] Kälin RE, Glass R. APLN/APLNR signaling controls key pathological parameters of glioblastoma. Cancers (Basel). 2021;13:3899. 34359800 10.3390/cancers13153899 PMC8345670

[15] Wang Y, Wang G, Liu X, Yun D, Cui Q, Wu X, et al. Inhibition of APLN suppresses cell proliferation and migration and promotes cell apoptosis in esophageal cancer cells <em>in vitro</em>, through activating PI3K/mTOR signaling pathway. Eur J Histochem. 2022;66:3336. 35920446 10.4081/ejh.2022.3336 PMC9422863

[16] Zhou Y, Xu R, Luo J, Li X, Zhong Y, Sun Z. Dysregulation of miR-204-5p/APLN axis affects malignant progression and cell stemness of esophageal cancer. Mutat Res. 2022;825:111791. 35930907 10.1016/j.mrfmmm.2022.111791

[17] Zhu Y, Zhang P, Huo X, Ling Y, Lv X, Lin S, et al. Single-cell and spatial transcriptomics reveal apelin/APJ pathway’s role in microvessel formation and tumour progression in hepatocellular carcinoma. J Cell Mol Med. 2024;28:e70152. 39434201 10.1111/jcmm.70152 PMC11493554

[18] Lin TH, Chang SL, Khanh PM, Trang NTN, Liu SC, Tsai HC, et al. Apelin promotes prostate cancer metastasis by downregulating TIMP2 via increases in miR-106a-5p expression. Cells. 2022;11:3285. 36291151 10.3390/cells11203285 PMC9600532

[19] Altinkaya SO, Nergiz S, Küçük M, Yüksel H. Apelin levels are higher in obese patients with endometrial cancer. J Obstet Gynaecol Res. 2015;41:294–300. 25160885 10.1111/jog.12503

[20] Hu D, Cui Z, Peng W, Wang X, Chen Y, Wu X. Apelin is associated with clinicopathological parameters and prognosis in breast cancer patients. Arch Gynecol Obstet. 2022;306:1185–95. 35249152 10.1007/s00404-022-06433-3

[21] Wang Q, Wang B, Zhang W, Zhang T, Liu Q, Jiao X, et al. APLN promotes the proliferation, migration, and glycolysis of cervical cancer through the PI3K/AKT/mTOR pathway. Arch Biochem Biophys. 2024;755:109983. 38561035 10.1016/j.abb.2024.109983

[22] Yuan L, Jiang H, Jia Y, Liao Y, Shao C, Zhou Y, et al. Fatty acid oxidation supports lymph node metastasis of cervical cancer via acetyl-CoA-mediated stemness. Adv Sci (Weinh). 2024;11:e2308422. 38520724 10.1002/advs.202308422 PMC11151054

[23] Hirschler-Laszkiewicz I, Chen SJ, Bao L, Wang J, Zhang XQ, Shanmughapriya S, et al. The human ion channel TRPM2 modulates neuroblastoma cell survival and mitochondrial function through Pyk2, CREB, and MCU activation. Am J Physiol Cell Physiol. 2018;315:C571–C586. 30020827 10.1152/ajpcell.00098.2018 PMC6230687

[24] Lesiak A, Pelz C, Ando H, Zhu M, Davare M, Lambert TJ, et al. A genome-wide screen of CREB occupancy identifies the RhoA inhibitors Par6C and Rnd3 as regulators of BDNF-induced synaptogenesis. PLoS ONE. 2013;8:e64658. 23762244 10.1371/journal.pone.0064658 PMC3675129

[25] Ishimaru Y, Shibagaki F, Yamamuro A, Yoshioka Y, Maeda S. An apelin receptor antagonist prevents pathological retinal angiogenesis with ischemic retinopathy in mice. Sci Rep. 2017;7:15062. 29118394 10.1038/s41598-017-15602-3 PMC5678128

[26] Zhang M, Li Y, Wang H, Yu W, Lin S, Guo J. LncRNA SNHG5 affects cell proliferation, metastasis and migration of colorectal cancer through regulating miR-132-3p/CREB5. Cancer Biol Ther. 2019;20:524–36. 30395767 10.1080/15384047.2018.1537579 PMC6422517

[27] Kuonqui K, Campbell AC, Sarker A, Roberts A, Pollack BL, Park HJ, et al. Dysregulation of lymphatic endothelial VEGFR3 signaling in disease. Cells. 2023;13:68. 38201272 10.3390/cells13010068 PMC10778007

[28] Korhonen EA, Murtomäki A, Jha SK, Anisimov A, Pink A, Zhang Y, et al. Lymphangiogenesis requires Ang2/Tie/PI3K signaling for VEGFR3 cell-surface expression. J Clin Invest. 2022;132:e155478. 35763346 10.1172/JCI155478 PMC9337826

[29] Trang NTN, Lai CY, Tsai HC, Huang YL, Liu SC, Tsai CH, et al. Apelin promotes osteosarcoma metastasis by upregulating PLOD2 expression via the Hippo signaling pathway and hsa_circ_0000004/miR-1303 axis. Int J Biol Sci. 2023;19:412–25. 36632453 10.7150/ijbs.77688 PMC9830518

[30] Kim JD, Kang Y, Kim J, Papangeli I, Kang H, Wu J, et al. Essential role of Apelin signaling during lymphatic development in zebrafish. Arterioscler Thromb Vasc Biol. 2014;34:338–45. 24311379 10.1161/ATVBAHA.113.302785 PMC3977740

[31] Berta J, Hoda MA, Laszlo V, Rozsas A, Garay T, Torok S, et al. Apelin promotes lymphangiogenesis and lymph node metastasis. Oncotarget. 2014;5:4426–37. 24962866 10.18632/oncotarget.2032 PMC4147335

[32] Peng X, Li F, Wang P, Jia S, Sun L, Huo H. Apelin-13 induces MCF-7 cell proliferation and invasion via phosphorylation of ERK1/2. Int J Mol Med. 2015;36:733–8. 26135903 10.3892/ijmm.2015.2265

[33] Mastrella G, Hou M, Li M, Stoecklein VM, Zdouc N, Volmar MNM, et al. Targeting APLN/APLNR improves antiangiogenic efficiency and blunts proinvasive side effects of VEGFA/VEGFR2 blockade in glioblastoma. Cancer Res. 2019;79:2298–313. 30718358 10.1158/0008-5472.CAN-18-0881

[34] Chen Y, Xu T, Xie F, Wang L, Liang Z, Li D, et al. Evaluating the biological functions of the prognostic genes identified by the Pathology Atlas in bladder cancer. Oncol Rep. 2021;45:191–201. 33200223 10.3892/or.2020.7853 PMC7709834

