# Supplementary material for: CREB5 promotes nodal metastasis of cervical cancer by regulation of APLN-induced lymphangiogenesis
Source: Cell Death Discov. 2025 Oct 27;11:488. doi: 10.1038/s41420-025-02782-5 (PMC12559322; doi:10.1038/s41420-025-02782-5)
Supplement: Supplementary file 1 — supplementary figures and figure legends. [file 41420_2025_2782_MOESM1_ESM.docx]

**
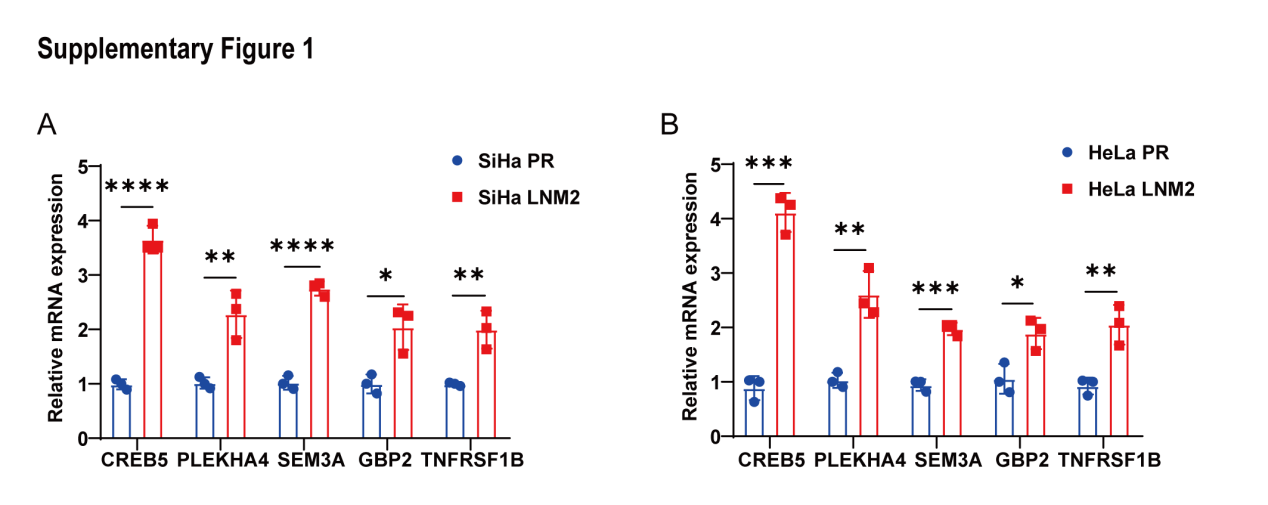
**

**Supplementary Figure 1. CREB5 is highly expressed in SiHa-LNM2 and HeLa-LNM2 cells.**

(A) RT-qPCR analysis of CREB5, PLEKHA4, SEM3A, GBP2, and TNFRSF1B expression in SiHa-PR and SiHa-LNM2 cells. (B) RT-qPCR analysis of CREB5, PLEKHA4, SEM3A, GBP2, and TNFRSF1B expression in HeLa-PR and HeLa-LNM2 cells.

**
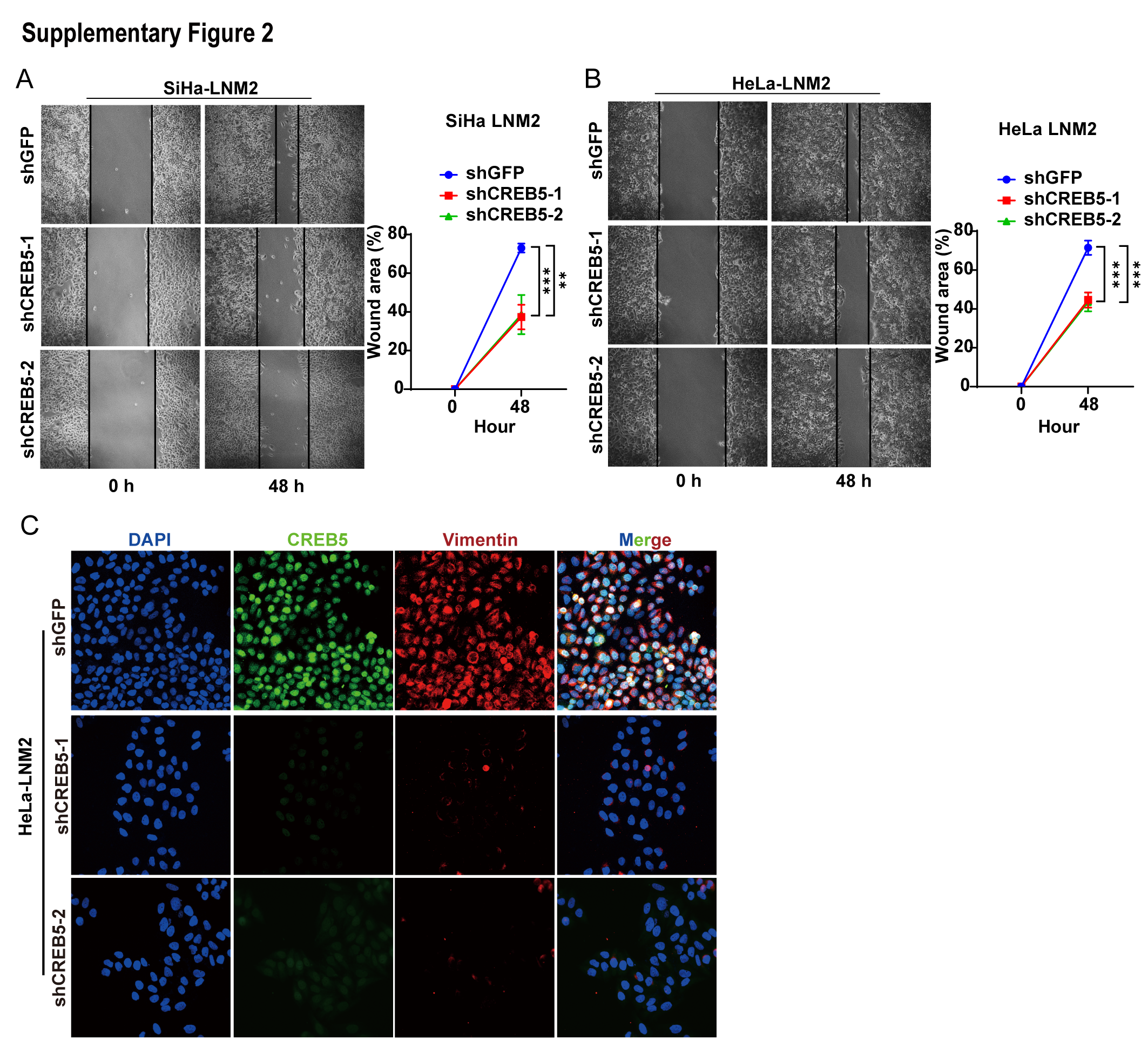
**

**Supplementary Figure 2. CREB5 promotes CCa cells migration and EMT *in vitro*.**

(A, B) Wound-healing assays were performed to investigate CREB5 knockdown suppressed migration of cervical cancer cells. (C) Representative IF images demonstrating that knockdown of CREB5 suppressed EMT in cervical cancer cells


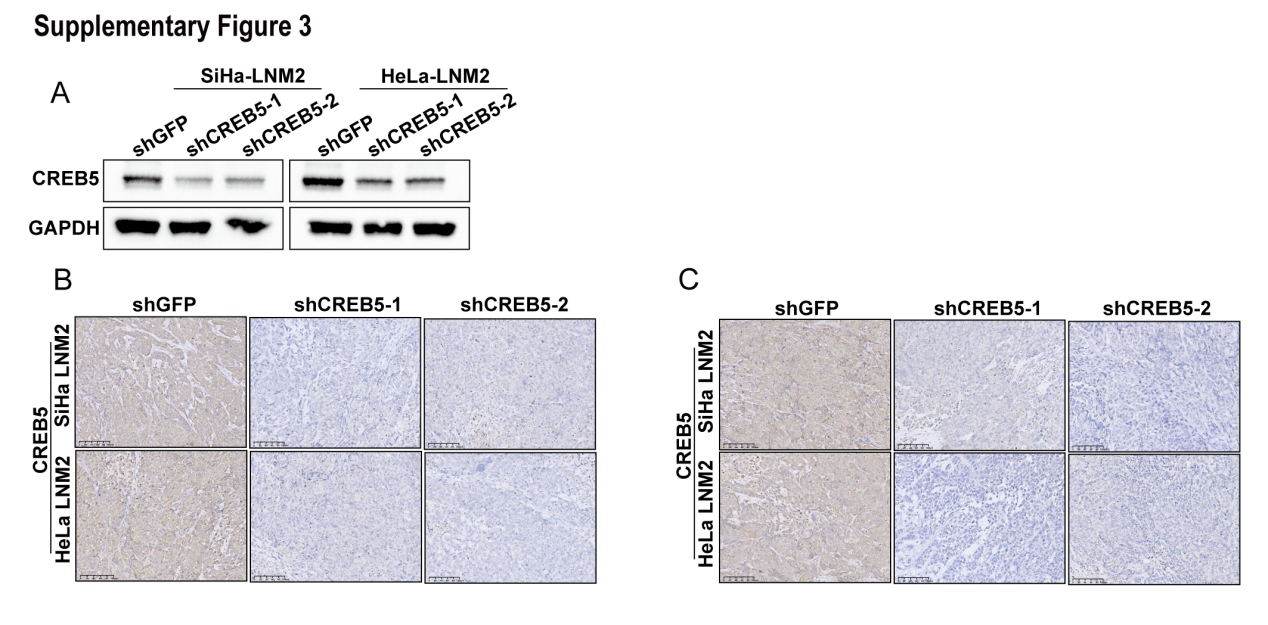
**Supplementary Figure 3. Validation of CREB5 knockdown efficiency in CCa cells and primary tumors.**

(A) Validation of CREB5 knockdown efficiency in CCa cells. (B, C) Representative IHC images demonstrating CREB5 knockdown efficiency in primary tumors.


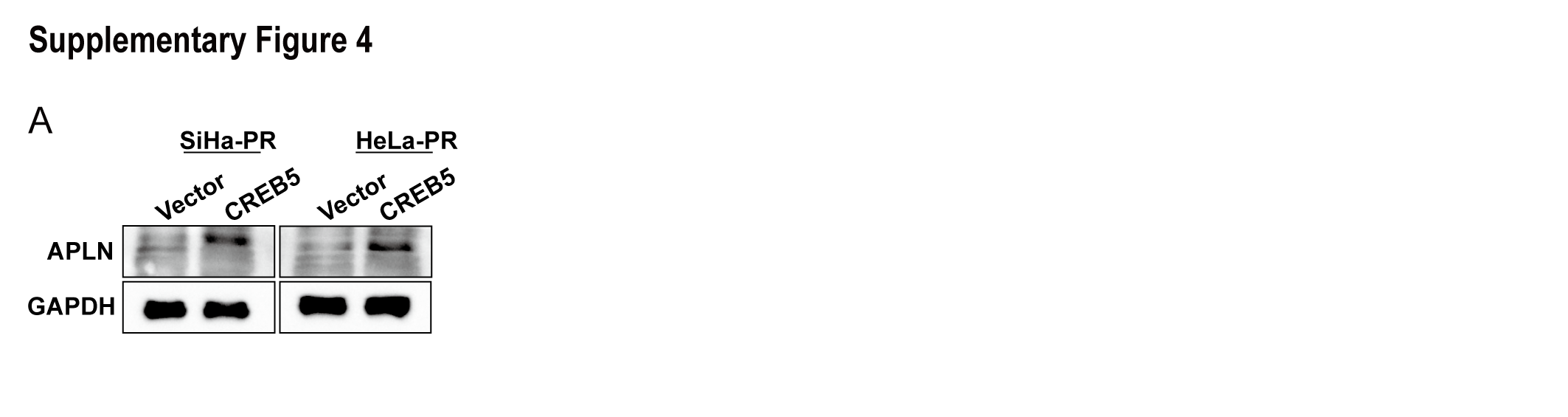


**Supplementary Figure 4. Overexpression of CREB5 increases APLN expression in CCa cells.**

(A) Western blot analysis of APLN protein levels in CCa cells with or without CREB5 overexpression.
