# Supplementary material for: CREB5 promotes nodal metastasis of cervical cancer by regulation of APLN-induced lymphangiogenesis
Source: Cell Death Discov. 2025 Oct 27;11:488. doi: 10.1038/s41420-025-02782-5 (PMC12559322; doi:10.1038/s41420-025-02782-5)
Supplement: Supplementary file 2 — supplementary Table S1 [file 41420_2025_2782_MOESM2_ESM.docx]

**Supplementary Table S1. Antibodies used in this study**

| CREB5 | Proteintech,#14196-1-AP | WB and IHC (1:1000)；IF (1:400) |
| --- | --- | --- |
| LYVE-1 | Abcam, #ab219556 | IF and IHC (1:2000) |
| D2-40 | MAB-0567 | IHC (1:400) |
| Pan-Cytokeratin | CST, #4545 | IHC (1:400), IF (1:500) |
| GAPDH | Proteintech,#10494-1-AP | WB (1:10000) |
| APLN | Active motif. #39133 | ChIP (10 µg per ChIP);  WB (1:1000) |
| Vimentin | Proteintech,#60330-1-Ig | IF (1:400) |
| AKT | CST, #4691 | WB (1:1000) |
| pAKT | CST, #4060 | WB (1:2000) |
| Donkey anti-Mouse IgG (H+L) Highly Cross-Adsorbed Secondary Antibody, Alexa Fluor™ 488 | ThermoFisher, #A21202 | IF (1:400) |
| Donkey anti-Rabbit IgG (H+L) Highly Cross-Adsorbed Secondary Antibody, Alexa Fluor™ 555 | ThermoFisher, #A31572 | IF (1:400) |
| HRP-conjugated Affinipure Goat Anti-Rabbit IgG(H+L) | Proteintech, #SA00001-2 | WB (1:10000) |
| HRP-conjugated Affinipure Goat Anti-Mouse IgG(H+L) | Proteintech, #SA00001-1 | WB (1:10000) |
