# Supplementary figures and images for: CREB5 promotes nodal metastasis of cervical cancer by regulation of APLN-induced lymphangiogenesis
Source: Cell Death Discov. 2025 Oct 27;11:488. doi: 10.1038/s41420-025-02782-5 (PMC12559322; doi:10.1038/s41420-025-02782-5)

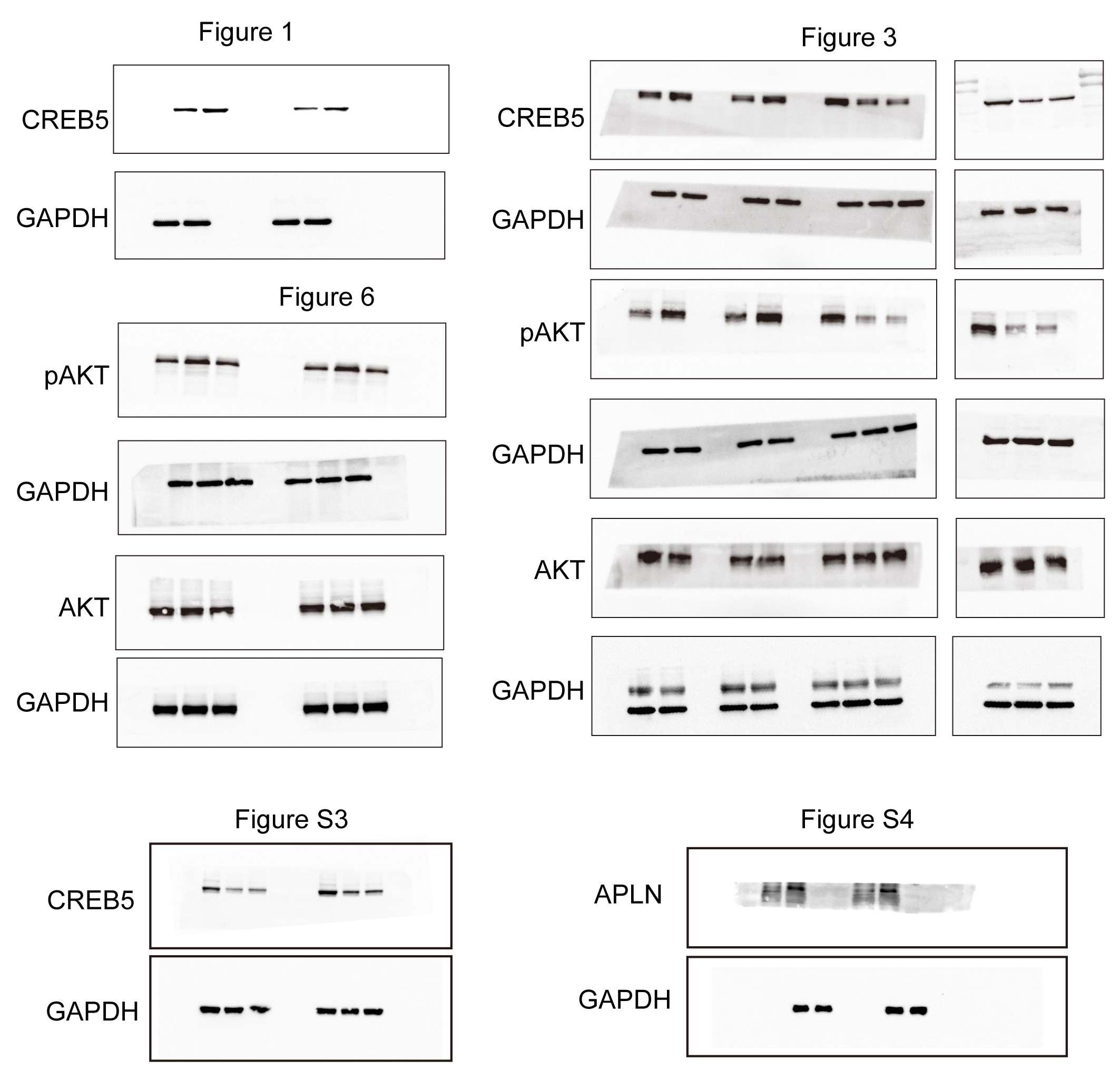

Supplement: Supplementary file 3 — uncropped blots [file 41420_2025_2782_MOESM3_ESM.tif]
